# High-quality faba bean reference transcripts generated using PacBio and Illumina RNA-seq data

**DOI:** 10.1038/s41597-024-03204-4

**Published:** 2024-04-09

**Authors:** Na Zhao, Enqiang Zhou, Yamei Miao, Dong Xue, Yongqiang Wang, Kaihua Wang, Chunyan Gu, Mengnan Yao, Yao Zhou, Bo Li, Xuejun Wang, Libin Wei

**Affiliations:** Department of Economic Crops, Jiangsu Yanjiang Institute of Agricultural Science, Nantong, China

**Keywords:** Plant breeding, Plant molecular biology

## Abstract

The genome of faba bean was first published in 2023. To promote future molecular breeding studies, we improved the quality of the faba genome based on high-density genetic maps and the Illumina and Pacbio RNA-seq datasets. Two high-density genetic maps were used to conduct the scaffold ordering and orientation of faba bean, culminating in an increased length (i.e., 14.28 Mbp) of chromosomes and a decrease in the number of scaffolds by 45. In gene model mining and optimisation, the PacBio and Illumina RNA-seq datasets from 37 samples allowed for the identification and correction 121,606 transcripts, and the data facilitated a prediction of 15,640 alternative splicing events, 2,148 lncRNAs, and 1,752 fusion transcripts, thus allowing for a clearer understanding of the gene structures underlying the faba genome. Moreover, a total of 38,850 new genes including 56,188 transcripts were identified compared with the reference genome. Finally, the genetic data of the reference genome was integrated and a comprehensive and complete faba bean transcriptome sequence of 103,267 transcripts derived from 54,753 uni-genes was formed.

## Background & Summary

Faba bean (*Vicia faba* L.) is an important leguminous crop with many uses and a high nutritional value^1–4^. Furthermore, it can be used as green manure, which can effectively improve soil fertility and play a vital role in the sustainable development of green agriculture^5,6^.

Studies on the genetics and genomics of faba beans have lagged behind those of other staple crops because of their large genome size^7,8^. Although the reference genome of faba bean was published in March 2023^9^, there are still many aspects that can be improved, such as the quality of the genome and analysis of gene structures and transcription factors. High-quality reference genomes are usually considered important resources for promoting genomic breeding programs and molecular investigations^10^. Genetic maps, Hi-C, long sequence fragments, and collinearity analysis with closely related species (e.g., soybean) can effectively improve the quality of the faba bean genome. PacBio single-molecule long-read transcriptome sequencing technology can not only discover new genes but also supplement the gene structure information of the genome.

**Fig. 1:**
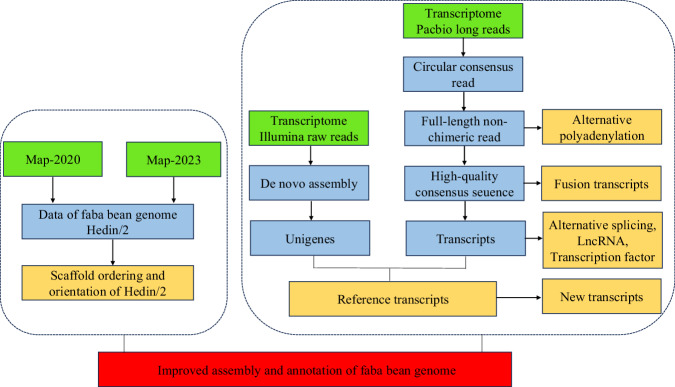
The workflow in this study. The panes with green, blue, yellow and red represent the input data, intermediate files, final outputs, final result respectively.

In this study (Fig. 1), according to our previous high-density genetic map (i.e., Map-2023)^11^ and the genetic map published by Carrillo-Perdomo in 2020 (i.e., Map-2020)^12^, we conducted the scaffold ordering and orientation of the faba bean genome. The published reference genome has a total length of 11.9 Gbp and contains 3,979 scaffolds^9^. During the scaffold ordering and orientation process, 13,121 markers provided valid information, and anchored a total amount of 11.28 Gbp sequences in chr1-chr6, accounting for 94.7% of the total length (Table 1). Compared to the previous reference genome, the length of genome increased 14.28 Mbp of chromosomes (chr1-chr6) and the short scaffolds number decreased by 45 (Table 2).

**Table 1:** Summary for consensus mapping in Chr1–6 of reference genome.

Item	Map-2020	Map-2023	Oriented	Two maps anchored	Unplaced
Markers (unique)	1,713	12,074	12,997	13,121	666
Markers per Mb	0.2	1.1	1.2	1.2	1.1
N50 Scaffolds	4	4	4	4	0
Scaffolds	86	228	13	52	3,934
Scaffolds with 1 marker	76	89	1	17	113
Scaffolds with 2 markers	2	48	2	9	45
Scaffolds with 3 markers	1	23	1	8	18
Scaffolds with > = 4 markers	7	68	9	18	52
Total bases (bp)	1,291,049,139 (94.8%)	11,331,352,125 (95.1%) (94.6%)	11,268,199,942 (94.7%)	11,280,740,986 (94.7%)	633,795,198 (5.3%)

**Table 2:** Comparison of the reference genome before and after scaffold ordering and orientation of the genetic map.

Item	Reference genome	Genome after scaffold ordering and orientation
Total length of Chr1–6	11.266 Gbp	11.281 Gbp
Scaffold number	3,979	3,934
Total length of Scaffold	648,070,065 bp	633,795,198 bp

**Fig. 2:**
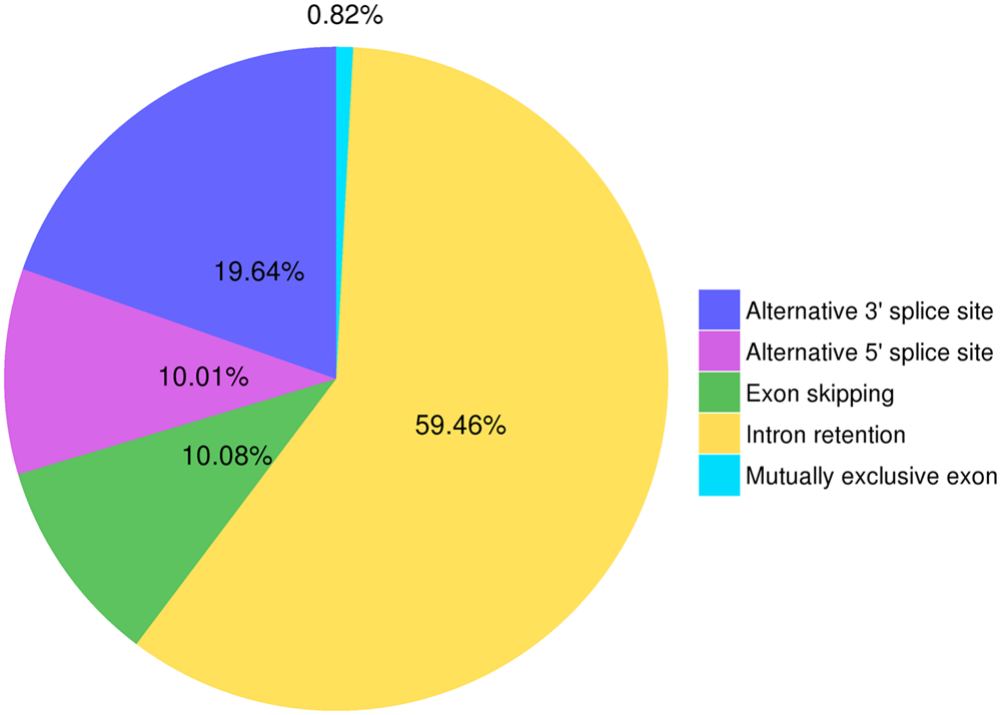
The summary of alternative splicing events. Pie chart showing the proportion of alternative 3′ splice site (blue), alternative 5′ splice site (purple), exon skipping (green), intron retention (yellow), mutually exclusive exon (light blue).

**Fig. 3:**
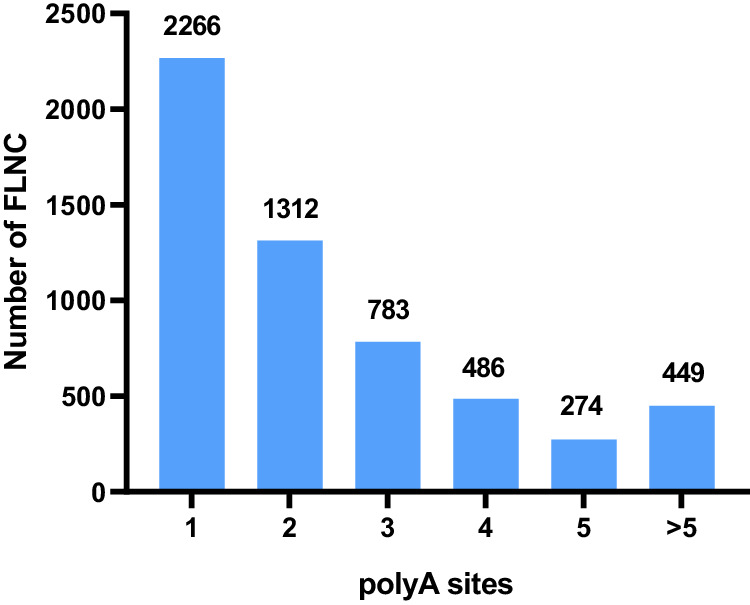
Statistics of alternative polyadenylation. The X axis represents the polyA sites number of alternative polyadenylation, and the Y axis represents the number of FLNC.

**Fig. 4:**
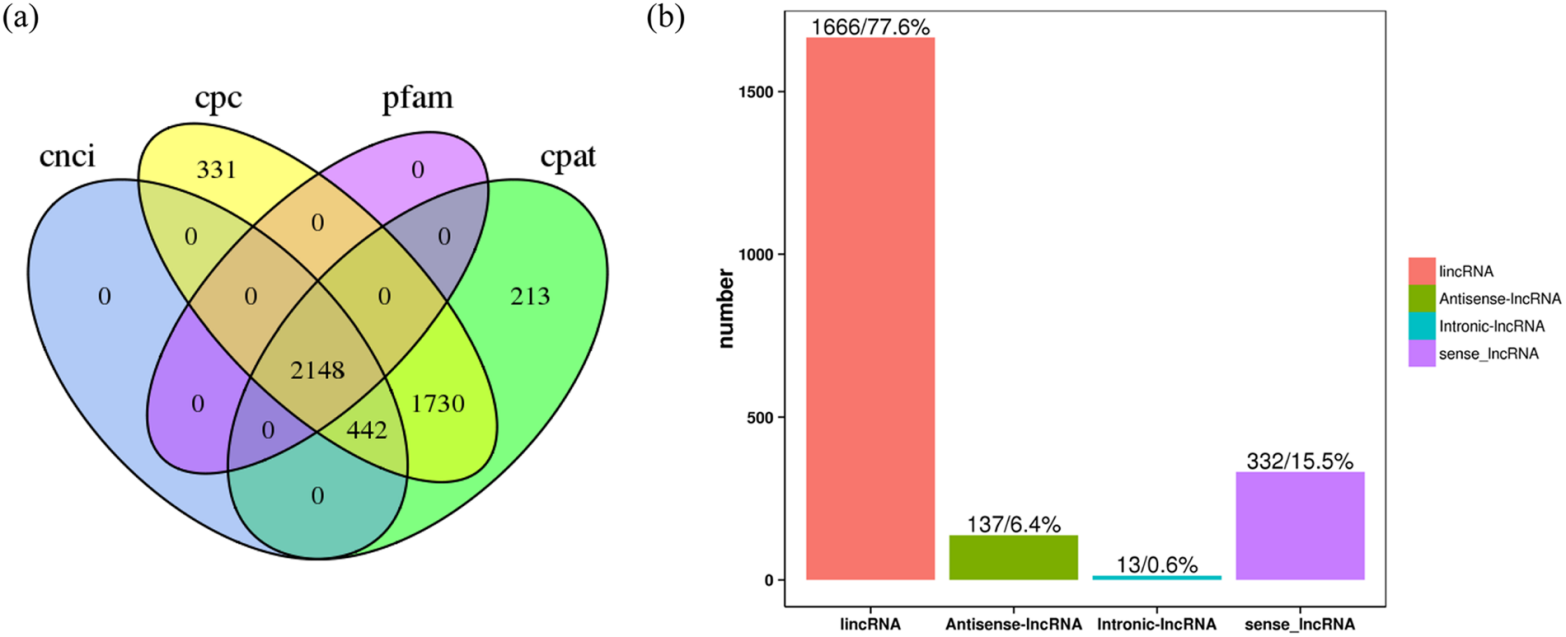
LncRNA prediction results. (**a**) Venn diagram of LncRNAs predicted by Coding Potential Calculator, Coding-Non-Coding Index, Coding Potential Assessment Tool and Pfam. (**b**) LncRNA position classification.

**Fig. 5:**
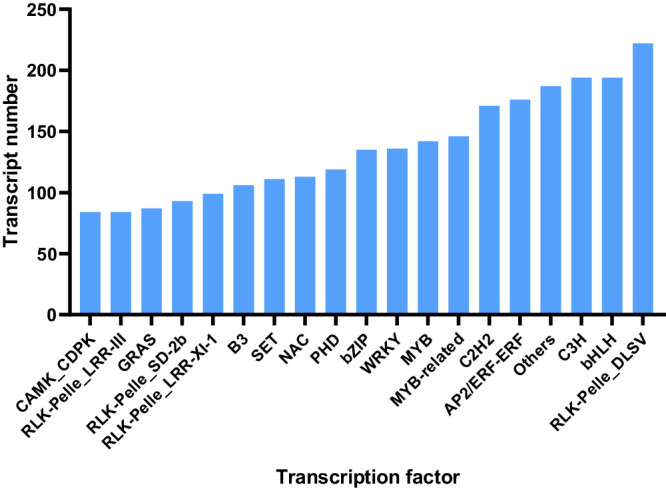
Statistics of transcription factor. The X axis represents the family type of transcription factor, and the Y axis represents the number of transcription factor family.

To facilitate functional genomic studies in faba beans, thirty seven different tissue samples were collected and fed to PacBio and Illumina for RNA sequencing, and 121,606 high-quality transcripts were harvested. And a total of 15,640 alternative splicing events (Fig. 2), 5,570 alternative polyadenylation (Fig. 3), 2,148 lncRNAs (Fig. 4), 1,752 fusion transcripts, and 6,568 transcription factors (Fig. 5) were also predicted.

Moreover, a total of 30,009 genes derived from the 121,606 transcripts above can fully or partially match with the genes of the reference genome, which covered 87.7% of the total number of genes (34,221 protein-coding genes in total) in the reference genome. More importantly, a total of 38,850 new genes loci including 56,188 transcripts were identified compared with the reference genome. In order to evaluate the protein-encoding potential of the new genes, we blasted their sequences to the protein-encoding genes from faba bean, chickpea and *M. truncatula*, and found that 33,910 transcripts from 19,063 genes can get a hit. By adding these new genes, the annotation completeness BUSCO score of the reference genome increased from 95.4% to 98.7%. In order to provide a comprehensive transcription information, the RNA-seq datasets above were integrated into the reference genome and a new annotation dataset consisting of 103,267 sequences and 54,753 uni-genes was harvested. Our study provides comprehensive reference genome information for faba beans.

## Methods

### Faba bean genome scaffold ordering and orientation by genetic map

The analysis entailed the use of the following data: a new high-density genetic map constructed by our group based on transcriptome sequencing (Map-2023), the genetic map from the previously published genome of faba bean by Carrillo-Perdomo (Map-2020), and the data of Hedin/2 from the newly published faba bean genome (http://w3lamc.umbr.cas.cz/lamc/?page_id=8, 2023). To anchor various genetic maps, the upstream and downstream sequences of the map markers (Map-2020) and (Map-2023) were first anchored to scaffolds in the reference genome using BLASTN, and the anchor points of the different markers were obtained according to the highest bitscore from blastn. A comparison of the two genetic maps with the reference genome showed that the two maps contained 1,713 and 12,074 marker sites. Map-2020 covered 86 scaffolds of the genome, accounting for 94.8% of the total length. Map-2023 covered 228 scaffolds, accounting for 95.1% of its total length (Table 1). Comparisons between the genetic map information and the physical map location of the markers were performed using Python scripts. Finally, the sequencing orientation and position of each scaffold in the reference genome were analyzed using ALLMAPS^13^, and the gap lengths among different scaffolds were estimated.

### Illumina and Pacbio-based RNA-seq

#### Sample collection and sequencing

The cultivars ‘QiDou2’, vf21376, vf21378 and vf301 were used for the transcriptome sequencing, which were planted in Jiangsu Yanjiang Institute of Agricultural Sciences, China, in Spring 2021. Full-length transcripts generated from different faba bean tissues using the PacBio and Illumina RNA-seq datasets were used for gene annotation. PacBio reads were generated from the total RNA of nine tissues which were mixed in equal amounts, including roots, leaves, flowers, seeds and pods at different developmental stages of cultivar ‘QiDou2’. The raw sub-reads were analyzed following the Iso-Seq 3 pipeline. Polished Circular Consensus (CCS) sub-reads were generated using CCS v6.2.0. Lima v2.1.0 and isoseq 3 refine were used to remove the primers and poly(A) tails, respectively. Full-length consensus sequences were mapped to reference genome using minimap2. Mapped reads were further collapsed by cDNA-Cupckae. BUSCO was used to analyze the completeness and accuracy. Thirty-six Illumina RNA-seq libraries were respectively generated from roots, leaves, flowers, seeds and pods of cultivar ‘QiDou2’, flowers of vf21376, vf21378 and vf301, then sequenced by Illumina NovaSeq 6000 sequencing platform (Biomarker technologies, Beijing, China). Clean reads were obtained by removing reads containing adapter, reads containing ploy-N and low-quality reads from raw reads. De novo assemblies were performed with these Illumina short reads using Trinity, then the blastn program was used to obtain high-quality integrated consensus sequences (121,606) from above PacBio and Illumina sequences. The analysis software and parameters are listed in Supplementary table 1. The materials and reads are listed in Supplementary table 2.

#### Structural analysis

A total of 15,640 alternative splicing events including 128 mutually exclusive exons, 9,299 intron retention, 1,576 exon skipping, 1,566 alternative 5′ splice sites and 3,071 alternative 3′ splice sites were identified by the AStalavista tool^14^ (Fig. 2). The alternative polyadenylation analysis and identification from full-length non-chimeric reads (FLNC) was conducted using TAPIS^15^ (Fig. 3).

#### LncRNA prediction

Coding Potential Calculator^16^, Coding-Non-Coding Index^17^, Coding Potential Assessment Tool^18^ and Pfam database were jointly applied to sort non-protein coding RNA candidates from putative protein-coding RNAs in the novel transcripts. A total of 2,148 lncRNA transcripts were acquired (Fig. 4), and the LncTar software^19^ was used to predict the RNA targets of the lncRNAs.

#### Fusion transcripts identification

Fusion transcripts were screened using the cDNA-Cupcake^20^ software. A total of 1,752 fusion transcripts were obtained.

#### Transcription factor analysis

The iTAK^21^ software was used to predict plant transcription factors, and 6,568 transcription factors were predicted (Fig. 5).

### Re-annotation of the reference genome

Minimap2^22^ was used to map our integrated consensus transcripts (121,606) to reference genome Hedin/2, and as a result, 74,959 genes, including 143,661 transcripts were annotated based on the mapping result. By comparing our annotation result with the original one of Hedin reference, a total of 30,009 genes can fully or partially (in “=ckmnjeosx” class code given by Gffcompare program^23^) match the genes of the reference genome, a total of 6,100 genes were aligned to the intron region of the reference genome, and with rest of 38,850 genes (in “u” class code) including 56,188 transcripts can not match with any gene of the reference genome, which were thus can be considered as new genes (Table 3). It was worthy note that the 38,850 new genes above include 2,148 lncRNAs. The 56,188 transcripts above, have 1–30 (1.93 on average) exons per transcript, and have a length of 82–8,425 bp (935.0 bp on average). By blast (blastx with evalue < 1E-10) these transcripts against the protein encoding sequences of faba bean, chickpea and Medicago, we found that 33,910 transcripts from 19,063 genes can get a hit, suggesting their protein encoding potential. After add these newly identified genes, the annotation completeness BUSCO score increased from 95.4% to 98.7%. In order to achieve a comprehensive and relative complete transcript dataset of the faba bean genome, the RNA dataset above, together with the CDS from the Hedin/2 reference original annotation, were fed to Cupcake ToFU collapsing pipeline, and as a result, 103,267 transcripts derived from 54,753 uni-genes were formed.

**Table 3:** Gene comparison type statistics of RNA-seq transcripts mapped to Hedin/2 genome.

Class code	Ref_gene number	Illumina + pacbio _gene number	Illumina + pacbio _transcripts number
=	24,494	23,733	26,409
c	2,106	2,095	2,478
e	1,743	1,731	1,928
i	3,858	5,469	6,741
j	7,360	7,165	13,670
k	3,640	3,534	6,367
m	5,886	5,801	10,547
n	3,872	3,797	6,328
o	3,480	3,302	4,948
p	1,737	1,763	2,049
s	753	764	1,041
u	—	38,850	56,188
x	3,718	3,948	4,782
y	145	143	185

**Note:** “ = ” represents complete, exact match of intron chain, “c” represents contained in reference (intron compatible), “e” represents single exon transfrag partially covering an intron, possible pre-mRNA fragment, “i” represents fully contained within a reference intron(s), “j” represents multi-exon with at least one junction match, “k” represents containment of reference (reverse containment), “m” represents retained intron(s), all introns matched or retained, “n” represents retained intron(s), not all introns matched/covered, “o” represents other same strand overlap with reference exons, “p” represents possible polymerase run-on(no actual overlap), “s” represents intron match on the opposite strand (likely a mapping error), “u” represents unknown, intergentic, “x” represents exonic overlap on the opposite strand(like “o” or “e” but on the opposite strand), “y” represents contains a reference within its intron(s). Different transcripts of the same gene may have different class_codes.

## Data Records

The PacBio and Illumina sequencing raw data cited in this work are stored in the National Center for Biotechnology Information (NCBI) under accession number SRP449779^24^. The datasets relating to the reference genome and the two genetic map can be obtained in references Jayakodi *et al*.^9^, Zhao *et al*.^11^, Carrillo-Perdomo *et al*.^12^. The high-quality integrated consensus transcripts (121,606) from PacBio and Illumina RNA-seq datasets are available from the NCBI with GenBank accession number GKNZ00000000^25^. Moreover, the comprehensive reference transcripts (103,267) and fusion genes (1,752) of faba bean generated in this study were downloaded from the NCBI with GKNU00000000 and GKNS00000000^25^, respectively. The files of genetic map mapped Hedin/2 and alternative splicing events, alternative polyadenylation, lncRNA, transcription factor are available at Figshare^26^.

## Technical Validation

### Linkage genetic map mounting

To ensure data accuracy, the self-constructed genetic map and the genetic map reported in 2020 were compared with the reference genome. Compared with the original genome, the improved genome increased the length of 14.28 Mbp and decreased the number of short scaffolds by 45.

### New gene mining

In this study, gene mining and optimisation were carried out in most steps. First, Illumina paired-end clean reads and PacBio long reads were mapped to the reference genome using minimap2. Second, redundancy was removed using cDNA_Cupcake to obtain more precise results. Finally, the collapsed isoform GTF files given by Cupcake were compared to the reference annotated transcripts using Gffcompare. Our data revealed 38,850 new genes compared with the reference genome. Moreover, the structure of the genes was analyzed, confirming that our work greatly improved the reference genome. The file of transcripts comparison between the Hedin reference and our generated sequences is available at Figshare^26^.

### Supplementary information

Supplementary table 1

Supplementary table 2

## Data Availability

No specific code was developed in this work. The parameters of bioinformatics tools and all software used for data processing were described in the Methods section and Supplementary table 1. If no detailed parameters are mentioned, the default parameters were used.
