# Supplementary material for: High-quality faba bean reference transcripts generated using PacBio and Illumina RNA-seq data
Source: Sci Data. 2024 Apr 9;11:359. doi: 10.1038/s41597-024-03204-4 (PMC11003973; doi:10.1038/s41597-024-03204-4)
Supplement: Supplementary file 1 — Supplementary table 1 [file 41597_2024_3204_MOESM1_ESM.doc]

**Supplementary table 1.** Tools used in analysis workflow with version and parameter.

| Tool | Version | Parameter | Description | URL |
| --- | --- | --- | --- | --- |
| ALLMAPS | v1.3.8 | Default | Computing a scaffold order that maximizes the colinearity to a collection of maps, including genetic, physical, or comparative maps into the final chromosome build | https://github.com/tanghaibao/jcvi/ |
| Astalavista | v3.2 | -t asta | Alternative splicing analysis | http://genome.imim.es/astalavista |
| Blastn | blast+2.14.0 | --task blastn-short, -evalue 1E-5, -outfmt 6 | Blast genetic map marker sequences to reference genome | https://blast.ncbi.nlm.nih.gov |
|  | 2.11.0 | -outfmt 6, -evalue 1e-5, -max_target_seqs 1, -max_hsps 1 | Obtain integrated consensus sequences from PacBio and Illumina RNA-seq datasets | https://bitbucket.org/comp_bio/tapis/overview |
| Blastx | blast+2.14.0 | evalue 1e-10 | Blast newly found CDS to other protein encoding sequences | https://blast.ncbi.nlm.nih.gov |
| BUSCO | v5.4.7 | -m ransciptome, -l endicots_odb10, --offline | Quantitative assessment of genome assembly and annotation completeness based on evolutionarily informed expectations of gene content. | https://busco.ezlab.org/ |
| ccs | v6.2.0 | --min-rq 0.9, --min-passes 3, -j 6, --min-length 200 | Obtain circular consensus read | https://www.pacb.com/support/software-downloads/ |
| cDNA_Cupcake collapse_isoforms_by_sam.py | 28.0.0 | -c 0.85, -i 0.9 | Collapse HQ isoform results to unique isoforms. | https://github.com/Magdoll/cDNA_Cupcake/wiki |
| cDNA_Cupcake fusion_finder.py | 28.0.0 | default | Find fusion genes | https://github.com/Magdoll/cDNA_Cupcake/wiki |
| CNCI | v2 | default | Predicting lncRNA | https://github.com/www-bioinfo-org/CNCI |
| CPAT | 1.2.2 | -cutoff 0.38 | Predicting lncRNA | https://sourceforge.net/projects/rna-cpat/files/v1.2.2/ |
| CPC2 | 0.1 | default | Predicting lncRNA | http://cpc2.gao-lab.org/ |
| Gffcompare | v0.9.8 | -G | Compare, merge, annotate and estimate accuracy of one or more GFF files | http://ccb.jhu.edu/software/stringtie/gffcompare.shtml |
| isoseq3 | v3.4.0 | refine, --require-polya | Obtain full-length non-chimeric reads | <https://github.com/PacificBiosciences/IsoSeq> |
| isoseq3 | v3.4.0 | Cluster, --verbose, --use-qvs | Obtain consensus transcripts by clustering | <https://github.com/PacificBiosciences/IsoSeq> |
| iTAK | v1.7a | default | Predicting transcription factor | http://itak.feilab.net/cgi-bin/itak/index.cgi |
| lima | v2.1.0 | --isoseq | Identify full-length reads | https://www.pacb.com/support/software-downloads/ |
| LncTar | v 1.0 | -d -0.1, -s F | Predicting the RNA targets of lncRNAs | http://www.cuilab.cn/lnctar |
| minimap2 | 2.20-r1061 | -ax splice, -uf, --secondary=no-C5 | Maping full-length consensus reads to reference genome | https://github.com/lh3/minimap2 |
| PfamScan | 1.60 | -translate orf | Predicting lncRNA | http://pfam.xfam.org/ |
| Python scripts | v1.0 | Default | Preparing the files for ALLMAPs from the blastn out | https://github.com/DrChunLi/home-build-python |
| SMRTLink | v10.1 | Default | Sequel/SequelII data toolset | https://www.pacb.com/support/software-downloads/ |
| TAPIS | 1.1.3 | default | Alternative polyadenylation analysis | https://bitbucket.org/comp_bio/tapis/overview |
| TransDecoder | v5.0.0 | -m 50, -Guniversal-S | Predicting coding sequence | https://github.com/TransDecoder/TransDecoder/releases |
| Trinity | v2.5.1 | Default | Illumina reads de novo assembly | https://github.com/trinityrnaseq/trinityrnaseq/wiki |
