# Supplementary material for: High-quality faba bean reference transcripts generated using PacBio and Illumina RNA-seq data
Source: Sci Data. 2024 Apr 9;11:359. doi: 10.1038/s41597-024-03204-4 (PMC11003973; doi:10.1038/s41597-024-03204-4)
Supplement: Supplementary file 2 — Supplementary table 2 [file 41597_2024_3204_MOESM2_ESM.doc]

**Supplementary table 2.** Details of materials and reads of PacBio and Illumina RNA-seq.

| Sequencing library | Library type | SRA Accession | Bases (Gbp) |
| --- | --- | --- | --- |
| PB full length for Pacbio-1 | Pacbio | SRX21042612 | 53.8G |
| root for Transcriptome-1 | Illumina | SRX21042615 | 6.1G |
| root for Transcriptome-2 | Illumina | SRX21042616 | 6.2G |
| root for Transcriptome-3 | Illumina | SRX21042617 | 6.2G |
| leaf for Transcriptome-1 | Illumina | SRX21042605 | 6.6G |
| leaf for Transcriptome-2 | Illumina | SRX21042613 | 6.8G |
| leaf for Transcriptome-3 | Illumina | SRX21042614 | 7.3G |
| flower for Transcriptome-1 | Illumina | SRX21042582 | 6.8G |
| flower for Transcriptome-2 | Illumina | SRX21042583 | 7.2G |
| flower for Transcriptome-3 | Illumina | SRX21042594 | 6.5G |
| seed-15d Transcriptome-1 | Illumina | SRX21042618 | 6.9G |
| seed-15d Transcriptome-2 | Illumina | SRX21042584 | 7.8G |
| seed-15d Transcriptome-3 | Illumina | SRX21042585 | 7.2G |
| seed-25d Transcriptome-1 | Illumina | SRX21042589 | 6.8G |
| seed-25d Transcriptome-2 | Illumina | SRX21042590 | 6.8G |
| seed-25d Transcriptome-3 | Illumina | SRX21042591 | 5.8G |
| seed-35d Transcriptome-1 | Illumina | SRX21042596 | 6.2G |
| seed-35d Transcriptome-2 | Illumina | SRX21042597 | 6.1G |
| seed-35d Transcriptome-3 | Illumina | SRX21042598 | 6.8G |
| pod-15d Transcriptome-1 | Illumina | SRX21042586 | 6.0G |
| pod-15d Transcriptome-2 | Illumina | SRX21042587 | 6.3G |
| pod-15d Transcriptome-3 | Illumina | SRX21042588 | 6.8G |
| pod-25d Transcriptome-1 | Illumina | SRX21042592 | 6.6G |
| pod-25d Transcriptome-2 | Illumina | SRX21042593 | 6.3G |
| pod-25d Transcriptome-3 | Illumina | SRX21042595 | 6.1G |
| pod-35d Transcriptome-1 | Illumina | SRX21042599 | 6.8G |
| pod-35d Transcriptome-2 | Illumina | SRX21042600 | 6.2G |
| pod-35d Transcriptome-3 | Illumina | SRX21042601 | 6.1G |
| purple flower for Transcriptome-1 | Illumina | SRX21042602 | 6.2G |
| purple flower for Transcriptome-2 | Illumina | SRX21042603 | 6.5G |
| purple flower for Transcriptome-3 | Illumina | SRX21042604 | 6.4G |
| white flower for Transcriptome-1 | Illumina | SRX21042606 | 6.2G |
| white flower for Transcriptome-2 | Illumina | SRX21042607 | 6.1G |
| white flower for Transcriptome-3 | Illumina | SRX21042608 | 6.1G |
| red flower for Transcriptome-1 | Illumina | SRX21042609 | 6.1G |
| red flower for Transcriptome-2 | Illumina | SRX21042610 | 6.1G |
| red flower for Transcriptome-3 | Illumina | SRX21042611 | 6.5G |
